# One-Step Electrochemical Synthesis of AlO_*x*_-Passivated Twisted-Phosphorene Nanosheets for Potentially Stable Energy Storage Devices

**DOI:** 10.1021/acsanm.2c05589

**Published:** 2023-03-01

**Authors:** Bing Wu, Lukáš Děkanovský, Jan Luxa, Pradip Kumar Roy, Guorong Hou, Liping Liao, Jan Paštika, Zdenek Sofer

**Affiliations:** Department of Inorganic Chemistry, University of Chemistry and Technology Prague, Technická 5, 166 28 Prague 6, Czech Republic

**Keywords:** black phosphorus, aluminum oxide, electrochemical exfoliation and passivation, ambient stability, twist angle

## Abstract

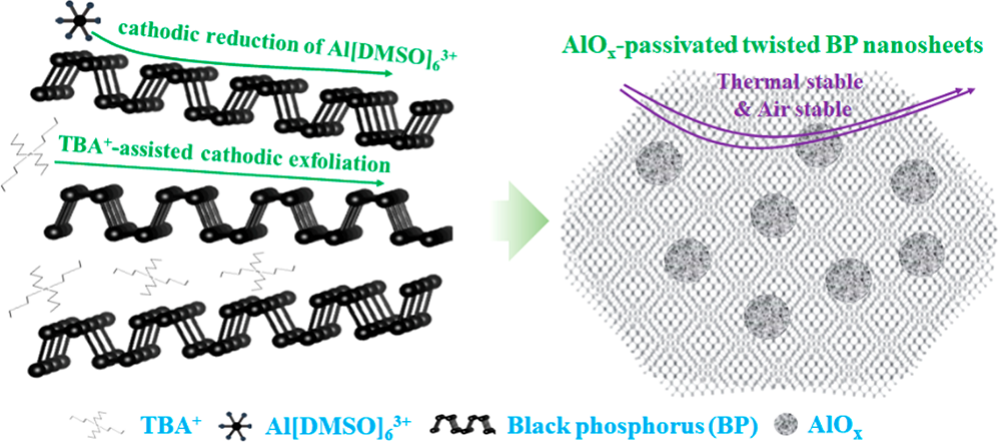

Black phosphorus (BP), a promising 2D material for electronics, energy storage, catalysis, and sensing, has sparked a research boom. However, exfoliated thin-layered BP is unstable and can easily be degraded under environmental conditions, severely limiting its practical applications. In this context, a simple and cost-effective method has been proposed that involves electrochemically exfoliating BP and simultaneously electrochemically depositing aluminum oxide (AlO_*x*_) for passivation of the exfoliated BP. The ambient stability of the exfoliated BP is studied using a time-dependent atomic force microscope (AFM). The AlO_*x*_ capping layer significantly improves the environmental stability of BP compared to uncapped BP. The thermal stability of the resulting BP is evaluated using power-dependent Raman spectroscopy. The results show that the AlO_*x*_-passivated BP has increased thermal stability, with only a slight shift in peak position toward higher Raman power intensity. These properties can make the material suitable for stable energy storage devices. Interestingly, the electrochemical exfoliation and passivation processes resulted in the BP with a twist angle (9.86°), which is expected to exhibit unique electronic properties similar to those of graphene with a twist angle.

## Introduction

Since the first exfoliation of graphene in 2004,^1^ intense research interest has been triggered in the development of other novel 2D materials, such as transition-metal dichalcogenides (TMDs)^2^ and Mxenes.^3^ Black phosphorus (BP), an emerging star of the 2D family, has drawn increasing attention due to its unique thermal and electronic properties for various applications in electronics, energy storage, catalysis, sensing, and antibacterial.^4−7^ BP is held together by van der Waals forces with a graphene-like layered hexagonal structure. However, it exhibits a reduced in-plane symmetry, with orthorhombic zigzag and armchair orientations caused by the nonequivalent sp^3^ hybridization of each P atom.^4^ Unlike graphene, BP is a semiconductor and exhibits a thickness-dependent band gap of 0.3 eV for the bulk to 2.0 eV for a monolayer,^8^ bridging the energy gap between graphene and TMDCs;^9^ it has several other properties, including an ultrahigh hole mobility of up to 10^3^ cm^2^ V^–1^ s^–1^ and a on/off current ratio of up to 10^5^ at room temperature.^10,11^

However, BP is unstable and prone to degradation, mainly from the continuous oxidation and hydration triggered by the surface P atoms under ambient conditions. This would significantly limit the practical applications of BP. Numerous methods have been used to protect BP, mainly by physical encapsulation and chemical treatments, such as deposition of an inorganic material,^4,12,13^ organic/inorganic functionalization, or surface passivation,^14−16^ some of which work well.^14^ To our knowledge, Al_2_O_3_-passivated few-layer BP prepared by atomic layer deposition (ALD) has been reported to be stable in the air for more than 100 h.^17^ Nevertheless, the challenge of controlling the quality of passivation layers remains unresolved. On the other hand, the ALD method is often costly, making it difficult to produce large quantities of samples.

In this work, a facile and cost-effective method, based on the electrochemical exfoliation of BP and synchronized electrochemical deposition of aluminum oxide (AlO_*x*_), was proposed to passivate exfoliated BP. The measured BP confirms that it is homogeneously decorated by aluminum oxide. We also show its stability in the environment by time-dependent AFM. Compared to the sample without passivation, the well-passivated BP offers stability in the air for over 120 h. Besides, the power-dependent Raman results suggest that the BP with a passivation layer exhibits higher thermal stability against increasing Raman power. The passivation processes we developed produce the BP with a stacked twist angle (∼9.86°), and it is expected that this finding will alter the electronic structure of BP to achieve surprising properties such as those of graphenes with twist angles.^17,18^ Due to its unstable nature, the application of black phosphorus (BP) in energy storage devices is often limited. We hope that this improved BP will facilitate its use in energy storage devices.

## Results and Discussion

### Properties of BP and BP-Al

**Figure 1:**
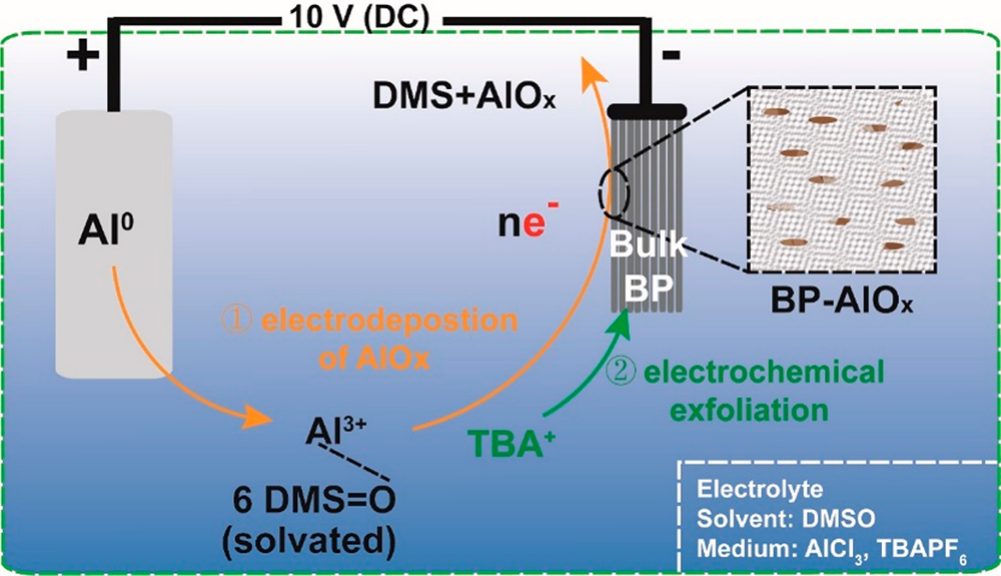
Schematical illustration of preparing AlO_*x*_-functionalized exfoliated black phosphorus.

The preparation of AlO_*x*_-functionalized exfoliated black phosphorus BP-Al is shown schematically in Figure 1. When a DC voltage is applied to the setup, the anode Al loses electrons and is oxidized into Al^3+^. The oxidized Al^3+^ continuously dissolves in the electrolyte and forms [Al(DMSO)_6_]^3+^ complexes on solvation with six DMSO molecules. On the cathodic side, some organic oxides, such as triphenylphosphine oxide (TPPO) and DMSO, can be electrochemically reduced to TPP and DMS using the Lewis acid AlCl_3_, as reported in the literature. Additionally, the oxygen atoms coordinated with aluminum form aluminum oxide: e.g. Al^3+^ + 6e^–^ + 3C_2_H_6_OS (DMSO) → Al_2_O_3_ + C_2_H_6_S (DMS)↑.^20,21^ In our designed experiments, the additional cation we introduced is [Al(DMSO)_6_]^3+^, which is expected to be reduced under bias voltage through the following reaction equation:
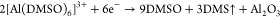
In the above reaction, DMSO is reduced to DMS, and at the same time, the Al connected to DMSO combines with the oxygen in DMSO to form aluminum oxide. This process can theoretically occur on both sides or between the interlayers of a few-layer BP. On the other hand, TBA^+^ (tetrabutylammonium ion) can be intercalated into the interlayer of bulk BP to obtain an exfoliated sample.^8,22^ The combination of these two substances is expected to exfoliate BP and, simultaneously, passivate the surface of the exfoliated BP with aluminum oxide.

**Figure 2:**
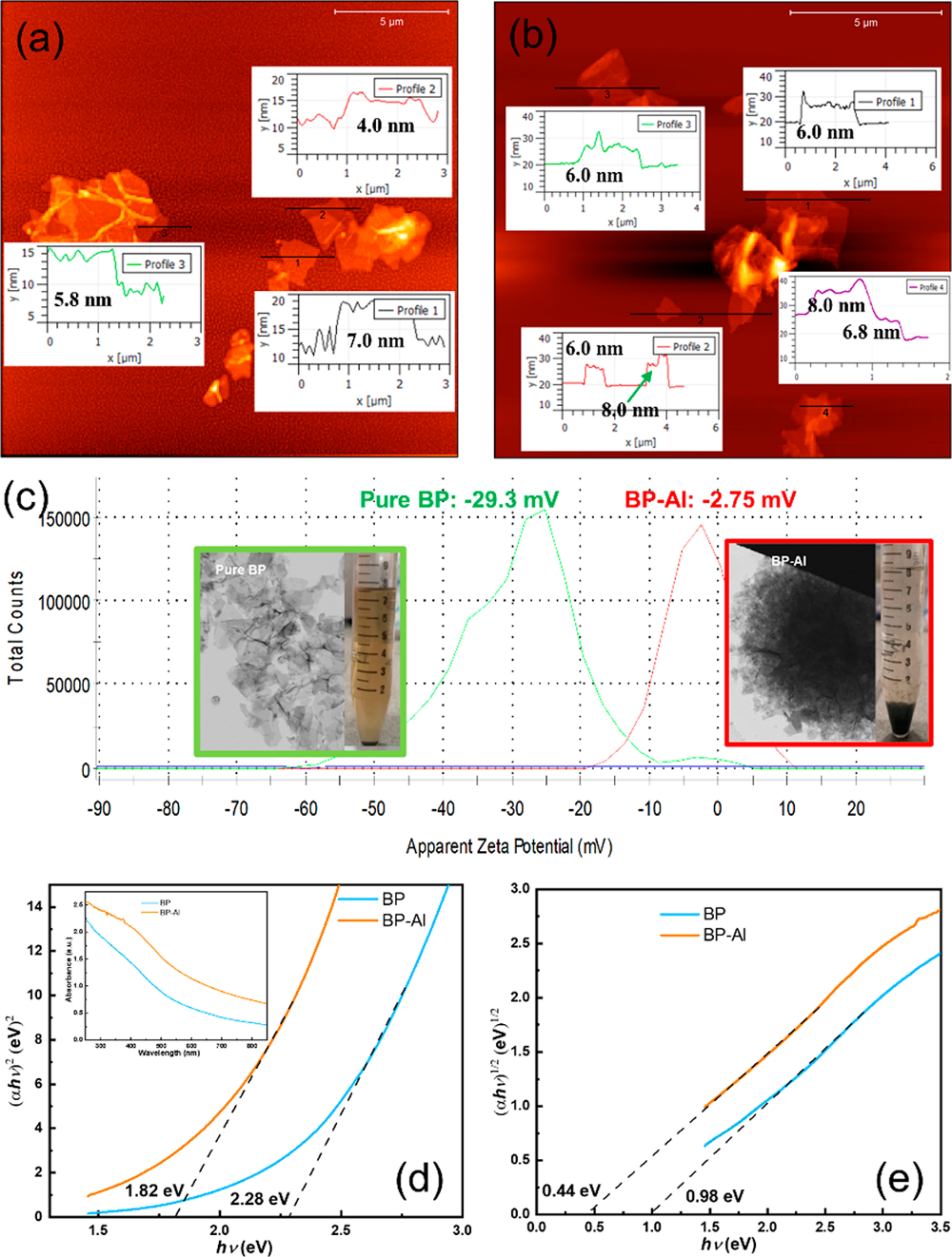
(a) AFM images of (a) BP and (b) BP-Al. (c) ζ potential comparison for BP and BP-Al in acetonitrile (insert: STEM images of BP and BP-Al and their photographs when they are dispersed in acetonitrile). (d) Tauc plot for direct band gap measurements (insert: corresponding UV–vis spectrum). (e) Tauc plot for indirect band gap measurements.

The thickness of electrochemically exfoliated BP samples is measured by AFM. As shown in Figure 2a,b, the randomly selected regions of AFM images displayed that BP-Al is slightly thinner than the sample without AlO_*x*_ decoration. Both BP and BP-Al are well exfoliated into few-layer nanosheets. The increased thickness of BP-Al might be ascribed to the surface decoration of Al-related species. All these 2D exfoliated BP samples were uniformly dispersed in acetonitrile at a concentration of 0.5 mg mL^–1^. As can be seen in Figure 2c, the ζ potential shows that both the TBA^+^-intercalated and the Al(DMSO)_6_]^3+^-assisted BP samples have a negative ζ potential. The value for BP is −29.3 mV and for BP-Al is −2.75 mV. Typically a , sample with a more negative ζ potential tends to show good long-term stability in solution, whereas particles with ζ values lower than −15 mV agglomerate rapidly if not sterically protected.^23^ As indicated by the inset images in Figure 2c, BP-Al is electronically unstable in ACN and tends to agglomerate, which might be caused by the attractive interactions between BP and the Al-related medium. The prepared BP nanosheets were further dispersed in water, alcohol, and NMP, as show in Figure S2. Interestingly, BP-Al has excellent hydrophilicity and can be well dispersed in water. In alcohol solvent, the AlO_*x*_-modified BP exhibits good dispersion and standing stability. In contrast, BP failed to stabilize in both water and alcohol. Similar to the phenomena in acetonitrile, BP-Al cannot be well dispersed in NMP, unlike the BP without modification. The absorption spectra of BP and BP-Al are measured to calculate their corresponding band gaps. Typically, black phosphorus is a direct band gap semiconductor from the monolayer to multilayers or bulk.^24^ As shown in Figure 2d, the band gaps calculated based on the direct band gap Tauc plot method are 1.82 and 2.28 eV for BP and BP-Al, respectively, significantly larger than reported theoretical band gap values of few-layer black phosphorus. For example, the monolayer BP has a direct band gap of ∼2.0 eV. Its band gap could be reduced to ∼1.0 eV for the three-layer structure and for the bulk to 0.3 eV.^25^ According to our AFM results, all prepared BP and BP-Al samples exhibit a thickness of over three layers and should have a bandgap below 1.0 eV, indicating the indirect band gap Tauc plot method in our results indicates a big difference with the reported BP band gap. Surface modifications by absorbing heteroatoms and solvent molecules can cause a transition from direct to indirect band gap in semiconductors. As a result, the band gap of the as-prepared material may also under a similar transformation from direct to indirect.^26−28^ Next, an indirect Tauc plot method was employed, as shown in Figure 2e, to determine the indirect band gap values of the prepared samples. The results exhibit more reasonable indirect band gap values of 0.98 eV for BP and 0.44 eV for BP-Al compared to the direct band gap Tauc plot method. Moreover, the obtained band gaps indicate that [Al(DMSO)_6_]^3+^-assisted exfoliation can reduce the band gap of BP. This phenomenon that the band gap of BP decreases after modification may be due to the conjugation effect between the BP and the surface-passivated AlO_*x*_,^29^ which needs to be validated through future theoretical calculations.

**Figure 3:**
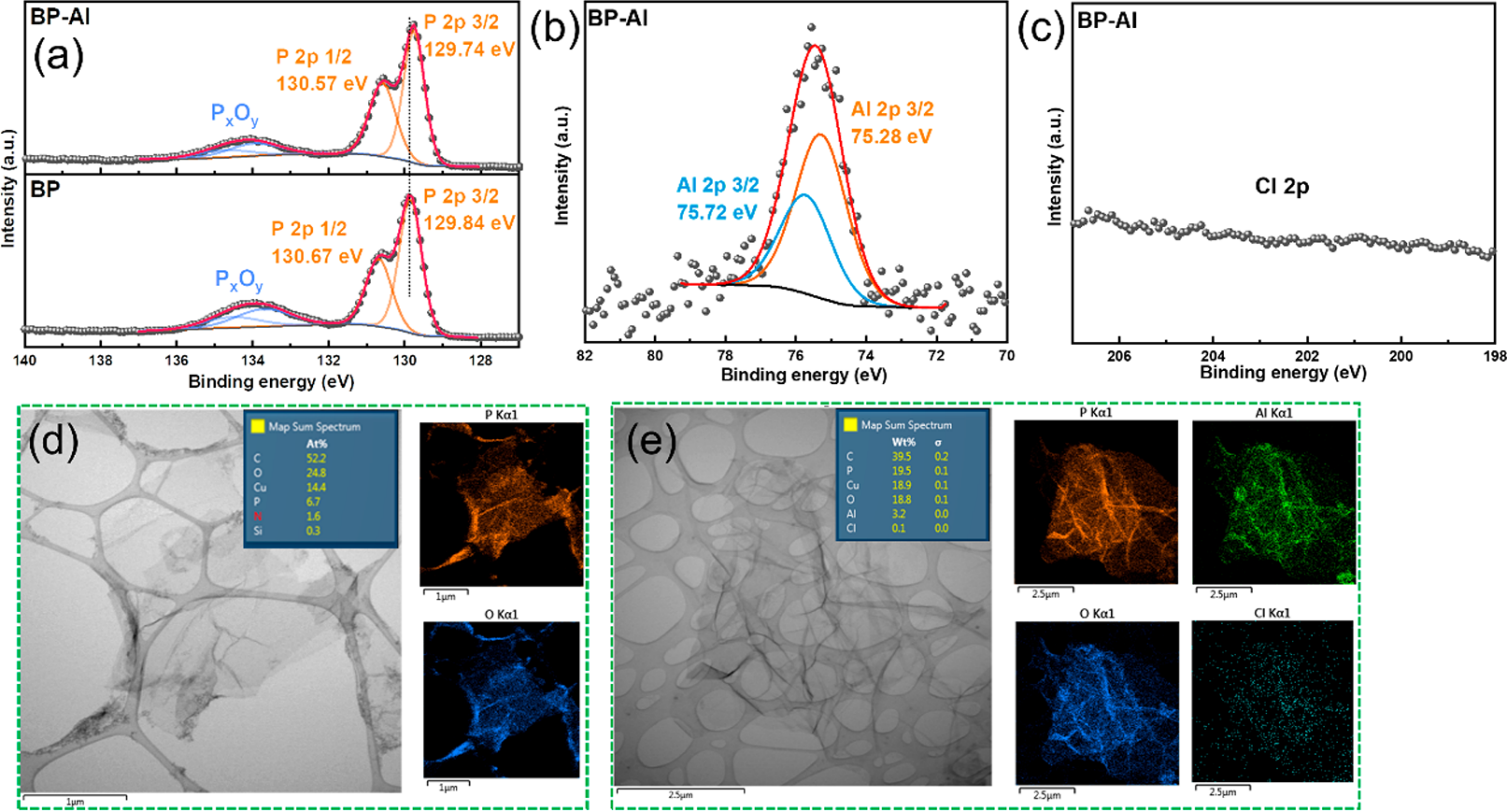
(a) XPS P 2p of BP and BP-Al and (b) Al 2p as well as (c) Cl 2p of BP-Al. STEM images (insert: elemental EDS analysis) and elemental mapping of (d) BP and (e) BP-Al.

The chemical compositions of as-prepared BP and BP-Al are evaluated by XPS. Figure 3a shows the P 2p_3/2_ and P 2p_1/2_ doublet at 129.84 and 130.67 eV for BP and at 129.74 and 130.57 eV for BP-Al, respectively, which are characteristics of the element phosphorus. The slight shift for BP-Al compared to BP is probably caused by the interaction between BP and Al-related compounds. The lower intensity of the oxidized phosphorus (P_*x*_O_*y*_) sub-band of ∼134.00 eV for BP-Al suggests that it has a stronger antioxidant ability than pure BP. Figure 3b shows Al 2p_3/2_ and Al 2p_1/2_ peaks at 75.28 and 75.72 eV, respectively, indicating an oxidized Al species. Recently, it was reported that the Lewis acid AlCl_3_ can naturally absorb on the surface of BP to passivate the BP.^14^Figure 3c shows no detectable Cl signals from the BP-Al. And the elemental EDS analysis in the inset of Figure 3e cannot detect Cl, in contrast to the high ratio of Al in BP-Al. These findings suggest that the Al species in the BP-Al sample is aluminum oxide (AlO_*x*_). The contrast STEM images and corresponding elemental mappings demonstrate that the prepared samples are composed of a few layers and the expected elements are uniformly distributed.

### Crystalline Structure

**Figure 4:**
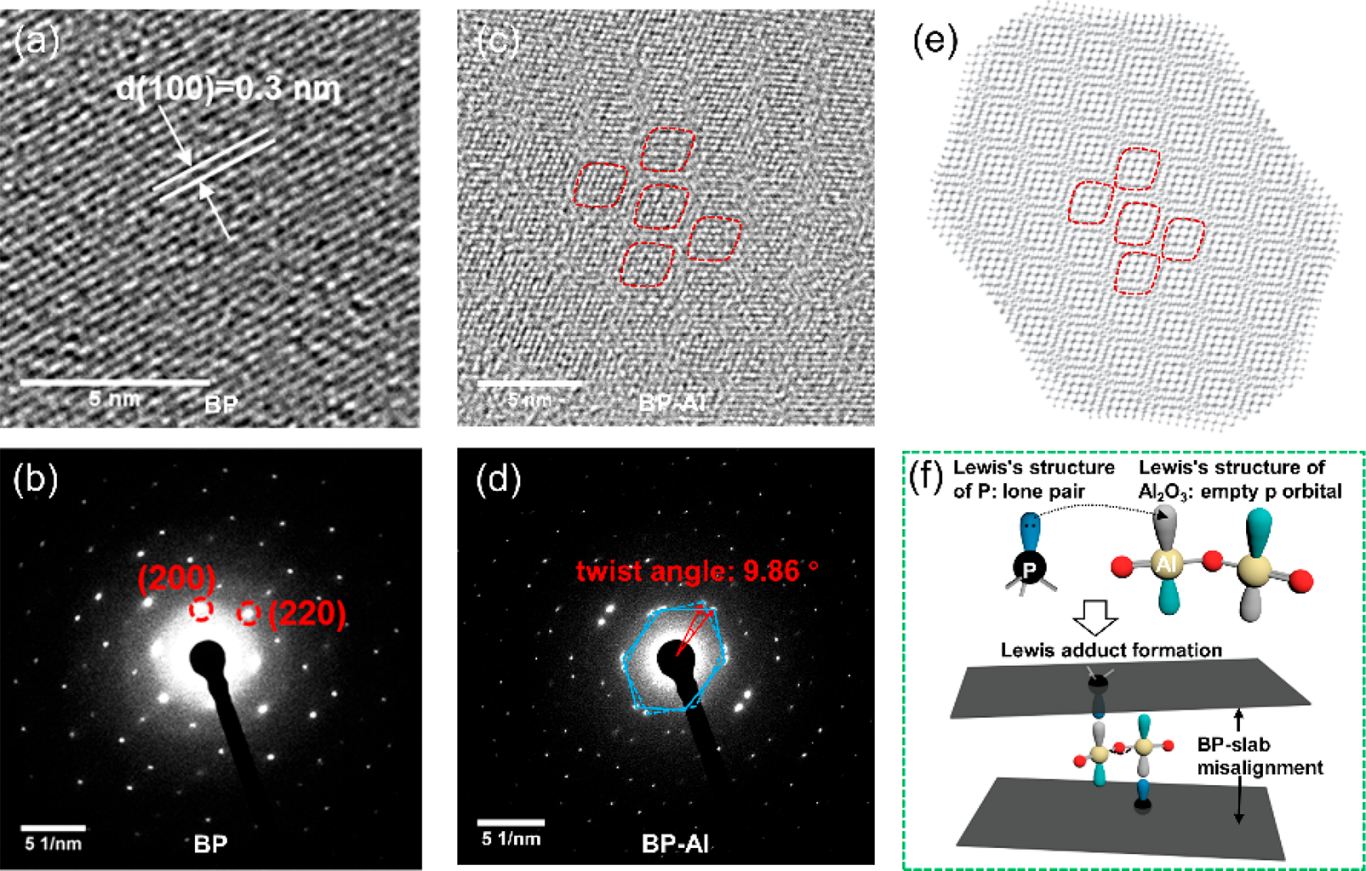
Sample BP: (a) HR-TEM and (b) SAED images. Sample BP-Al: (c) HR-TEM with Moiré fringes, (d) SAED with a twist angle, (e) simulated stacked monolayer BP with a twist angle of 9.86°, and (f) proposed model of the formation of stacked twist angle BP.

High-resolution TEM (HRTEM) and randomly selected area electron diffraction (SAED) are further employed to characterize the crystallinity of exfoliated samples. Figure 4a shows a clear lattice fringes of 0.3 nm, corresponding to the (100) planes of black phosphorus. The random SAED pattern in Figure 4b confirms that the exfoliated sample has a high-quality single-crystalline structure with an orthorhombic crystalline character. However, when the sample measurements were conducted for BP-Al, as shown in Figure 4c, the HRTEM image shows a Moiré fringe pattern caused by the misalignment of stacked BP. Figure 4d shows the SAED obtained from a random region of the TEM grid having a twist angle θ of 9.86°. Meanwhile, another SAED image in Figure S3 exhibits a mixture of twisted and nontwisted diffraction spots. Though our designed experiments failed to show a 100% yield of twisted BP flakes, it is still meaningful to promote the development of twisted 2D materials through a chemical-related synthesis strategy. Two monolayers BP with a twist angle of 9.86° are simulated, as shown in Figure 4e. The Moiré fringes in the simulated image match well with the HRTEM results in Figure 4c. To demonstrate the twist-angle formation of sample BP-Al, we propose the model shown in Figure 4f. The compound on the surface of BP is assumed to be Al_2_O_3_. Its Lewis structure features empty 3p orbitals in the valence band,^30^ which can accommodate the lone electron pairs of sp^3^ hybridization of P atoms in BP to form a Lewis adduct. The angle between two Al atoms might cause BP to be misaligned above and below Al_2_O_3_, leading to a unique pairing with the P elements. The X-ray diffraction of modified and not modified black phosphorus is shown on Figure S1. The dry suspension shows high preferential otentation and none crystaline Al_2_O_3_ phases.

### Ambient Stability

**Figure 5:**
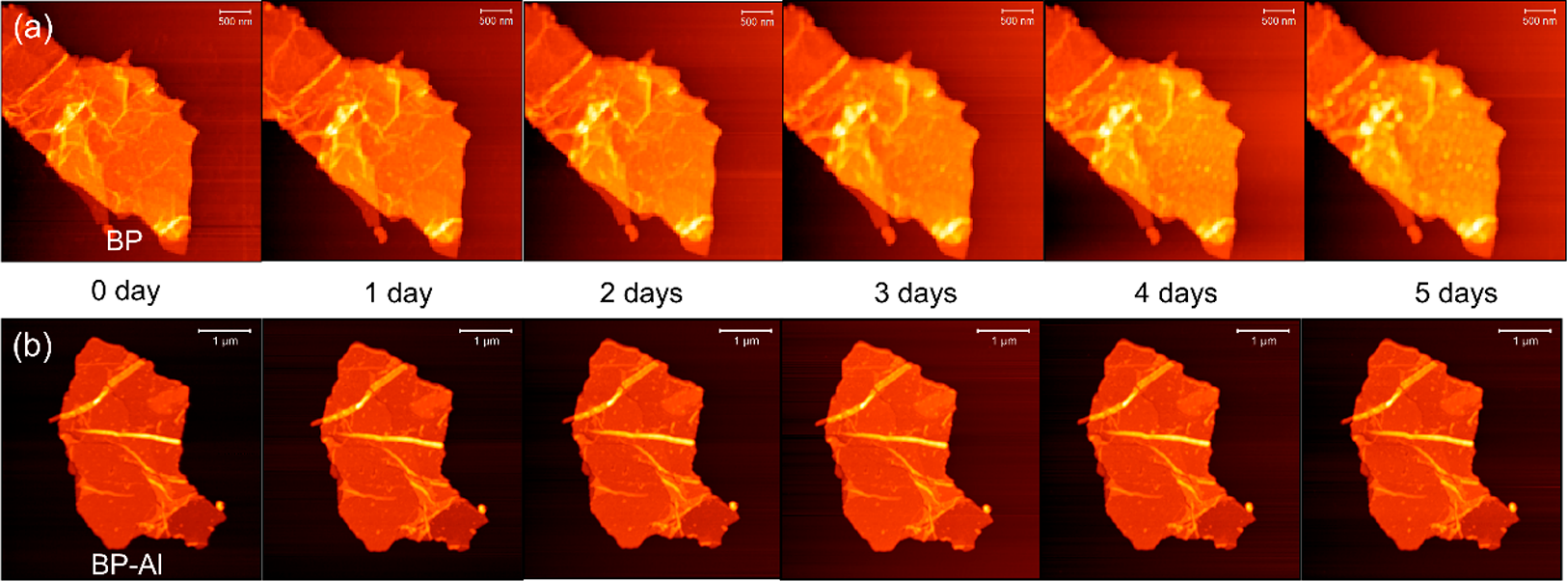
Time-dependent AFM images of (a) control and (b) AlO_*x*_-passivated BP samples.

The environmental stability of both pure BP and BP-Al was evaluated using time-dependent AFM topography under ambient conditions over a 5 day period. As shown in Figure S4, both samples had similar thicknesses of approximately 6 nm. Figure 5 displays the AFM images of the evolving topography of pure BP nanosheets and the AlO_*x*_-passivated BP over time. The AlO_*x*_-passivated BP showed exceptional environmental stability as compared to pure BP. No degradation of the flake was observed in the AlO_*x*_-passivated sample with increasing exposure time, while the pure BP nanosheet experienced degradation due to oxidation and water absorption, resulting in randomly increasing protrusions on the surface and unclear outlines of flakes.

In addition, STEM-EDS and Raman measurements were further conducted to study the stability of as-prepared samples. As shown in Figure S5, the BP nanosheet without AlO_*x*_ modification decomposed after 2 weeks of exposure to the ambient environment. In contrast, the AlO_*x*_-modified sample BP-Al still exhibited a clear nanosheet morphology. The time-dependent Raman spectra of BP and BP-Al in Figure S6 were collected to compare their antidegradation performance in the ambient environment. To reduce the effects of heating from the Raman laser on the samples, all the data were collected at a relatively low Raman laser intensity (0.5% of maximum). Obviously, with an increase in exposure duration, the peak intensity of BP gradually decreased while BP-Al exhibited a relatively strong Raman intensity after 5 days of exposure under ambient conditions. Those results further confirmed the excellent stability of as-prepared BP-Al.

### Thermal Stability

**Figure 6:**
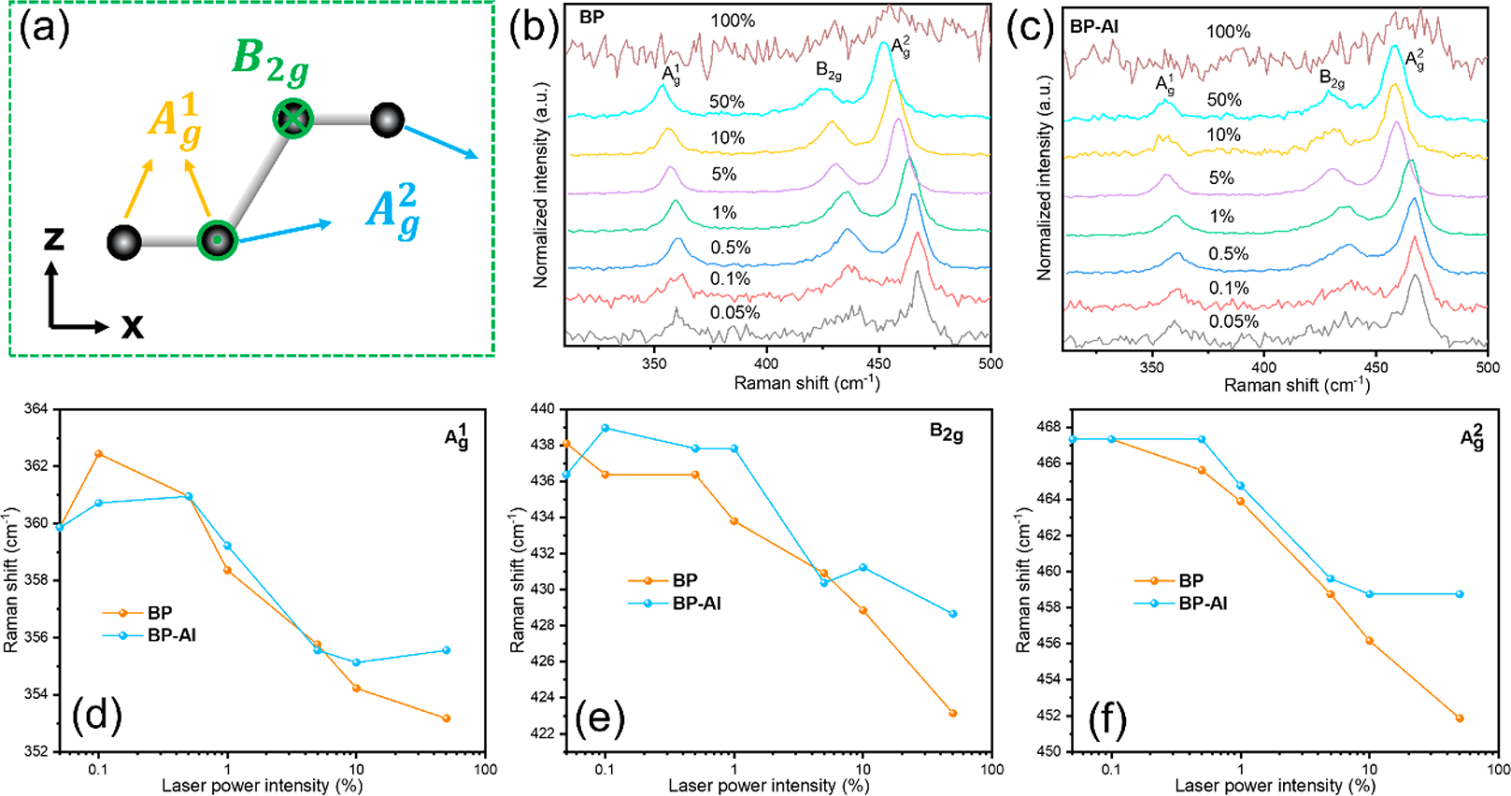
(a) Atomic displacement for three Raman modes of black phosphorus. Power-dependent Raman spectrum of BP (b) without and (c) with an AlO_*x*_ passivation layer with laser power intensities from 0.05% to 100%. (d–f) The A_g_^1^, B_2g_, and A_g_^2^ Raman mode frequencies of BP and BP-Al as a function of laser power intensity, respectively.

To investigate the thermal stability of BP, we carried out laser-power (which is positively associated with the heating temperature)-dependent studies on BP and BP-Al.^31^Figure 6a displays the three characteristic Raman modes from three directions of P–P bond vibrations of black phosphorus.^32^ As can be seen in Figure 6b,c, the peak positions of all three modes shifted with an overall increase in power, indicating an increase in the local temperature.^31^ Once the laser power intensity was increased to 100%, both samples were damaged, and the corresponding characteristic Raman modes disappeared. The frequency shift results are summarized and plotted in Figure 6d–f. Compared to the A_g_^2^ Raman mode, the red shifts below a laser intensity of 0.5% for A_g_^1^ and B_2g_ were irregular for both samples, ascribed to the error resulting from a lower signal for these two characteristic modes at low laser power. When the power intensity was increased from 0.5% to 50%, especially after 5% laser power intensity, BP without an AlO_*x*_ capping layer showed a more significant red shift than BP-Al. The results indicate that the AlO_*x*_-passivated BP effectively increases the thermal stability of black phosphorus.

## Conclusion

In summary, electrochemical exfoliation was combined with electrochemical deposition to produce aluminum oxide passivated black phosphorus (BP-Al). Results from STEM-EDS and XPS measurements showed that the BP-Al sample was well exfoliated and covered with aluminum oxide. Furthermore, the BP-Al sample displayed a restacked twist angle of 9.86° with regular Moiré fringes, which might be due to the unique pairing of aluminum with phosphorus that causes the misalignment of BP slabs and aluminum oxide below. Time-dependent AFM topography tests under ambient conditions and as a function of laser power revealed improved ambient and thermal stability in the BP-Al sample. This study presents an easy method for fabricating aluminum oxide–black phosphorus with good stability. Additionally, [Al(DMSO)_6_]^3+^-assisted electrochemical exfoliation used here resulted in BP with a unique twist angle, offering the potential for future exploration of its unique electronic properties, similar to those of graphene with twist angles. The improved air and thermal stability of the modified black phosphorus also indicates its further application in energy storage devices.

## Experimental Section

### Chemicals and Materials

Red phosphorus (99.999%), tin (99.999%), and AlCl_3_ (98%) were purchased from Sigma-Aldrich (Czech Republic). Iodine (99.9%), and dimethyl sulfoxide (DMSO) were obtained from Penta, Czech Republic. Carbon disulfide (CS_2_) (99.99%) was acquired from Acros Organics, Germany. Gold (99.99%) was acquired from Mateck, Germany. Tetrabutylammonium hexafluorophosphate (TBAPF_6_, 99%, powder) was purchased from Fluorochem. SnI_4_ was made by reacting Sn with I_2_ in chloroform under reflux and then recrystallizing the product.

Black phosphorus crystals were synthesized from red phosphorus as described previously.^19^ Briefly, red phosphorus and Sn/SnI_4_ (mineralizing agent) were placed in a quartz glass ampule and subsequently sealed using an oxygen/hydrogen torch. The ampule was placed horizontally in a muffle furnace and heated to 400 °C for 2 h. After that, the temperature was increased to 600 °C for another 24 h. Finally, the sample was slowly cooled to room temperature overnight. The obtained BP crystals with a metallic sheen were washed with CS_2_ to remove the byproduct of white phosphorus.

### Apparatus

The ζ potential was collected with Zetasizer ZSP instrument (Malvern Panalytical, England). A BLACK-Comet UV–vis spectrometer (StellarNet, United States) was used to record the visible light absorption spectra. Atomic force microscopy (AFM, Ntegra Spectra, NT-MDT) was conducted to measure the thickness of exfoliated BP nanosheets and their morphology changes under ambient exposure. Raman spectra were measured using an inVia Raman Microscope (Renishaw, England) in a backscattering geometry with a CCD camera detector and DPSS laser (532 nm, 50 mW at 100% as the power of laser source) and 20× objectives for the focusing of the sample. The thermal stability of exfoliated BP was evaluated through power-dependent Raman technology. The chemical composition and valence were analyzed by an X-ray photoelectron spectrometer (XPS, SPECS, Germany). Transmission electron microscopy (TEM, EFTEM Jeol 2200 FS microscope, Japan), equipped with energy dispersive spectroscopy (EDS, Oxford Instrument, England), was conducted to characterize the crystal structure, morphology, and elements.

### Sample Preparation

A facile two-electrode setup, controlled by an Auto PGSTA 204 instrument with NOVA 2.1 software (Metroohm, Switzerland), was used to exfoliate and functionalize BP as follows. A bulk BP crystal (∼50 mg) served as the cathode, while Al foil was employed as the anode. The solution, composed of solvent DMSO, 0.01 M TBAPF_6_, and 0.01 M AlCl_3_, was utilized as the supporting electrolyte. The parallel distance between Al foil and the BP crystal was about 2.5 cm, and a DC voltage at 10 V was used to trigger the exfoliation and functionalization. The prepared BP nanosheets were collected, washed with acetonitrile, and then dispersed into acetonitrile for later use. The functionalized sample was named BP-Al.

To determine the properties of functionalized BP, the same setup and processes as above were employed to prepare the nonfunctionalized pure BP. In this experiment, the anode Al was replaced by Pt and the composition of the supporting electrolyte was DMSO and 0.01 M TBAPF_6_. The prepared pure BP nanosheets were collected, washed with acetonitrile, and then dispersed into acetonitrile for later use. Pure BP nanosheets without AlO_*x*_ decoration was named BP.

## Supporting Information Available

XRD of exfoliated and treated BP, dispersion stability of modified BP flakes, TEM/EDS and SAED images of modified BP flakes, AFM images of modified BP flakes, and time-dependent Raman spectra of BP and modified BP (PDF)

## Abbreviations

EDS: energy-dispersive spectroscopy
TEM: transmission electron microscopy
STEM: scanning transmission electron microscopy
XPS: X-ray photoelectron spectroscopy
XRD: X-ray diffraction
ND: neutron diffraction
SAED: selected area electron diffraction
AFM: atomic force microscopy
DMSO: dimethyl sulfoxide
TBAPF_6_: tetrabutylammonium hexafluorophosphate
BP: black phosphorus
