# Supplementary material for: One-Step Electrochemical Synthesis of AlOx-Passivated Twisted-Phosphorene Nanosheets for Potentially Stable Energy Storage Devices
Source: ACS Appl Nano Mater. 2023 Mar 1;6(5):3912–8. doi: 10.1021/acsanm.2c05589 (PMC10018416; doi:10.1021/acsanm.2c05589)
Supplement: Supplementary file 1 — an2c05589_si_001.pdf [file an2c05589_si_001.pdf]

Supporting Information

One-Step Electrochemical Synthesis of AlO_x Passivated Twisted Phosphorene Nanosheets for Potentially Stable Energy Storage Device

Bing Wu, Lukas Dekanovsky, Jan Luxa, Pradip Kumar Roy, Guorong Hou, Liping Liao, Jan Paštika, Zdenek Sofer**

Department of Inorganic Chemistry, University of Chemistry and Technology Prague, Technická 5, 166 28 Prague 6, Czech Republic

* Zdeněk Sofer: zdenek.sofer@vscht.cz; * Bing Wu: wui@vscht.cz

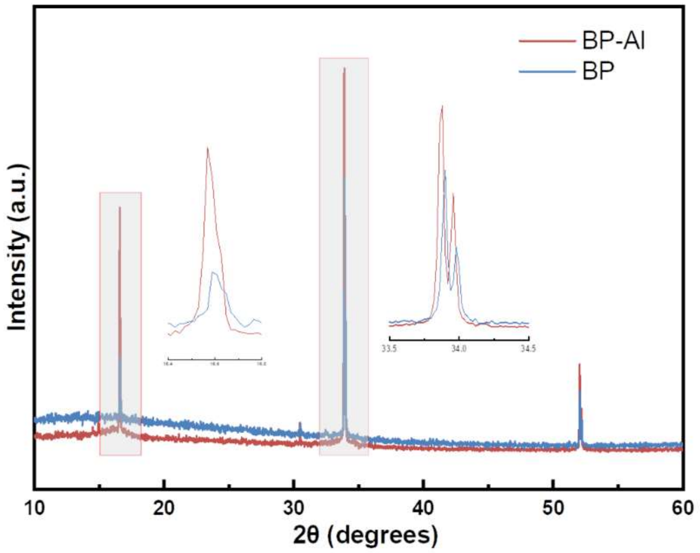

Figure S1. XRD patterns of BP-Al (red line) and BP (blue line). The sample alignment using a height (z) scan was performed to position the sample at the z height where half the direct beam intensity is attenuated, which can eliminate the influence of uneven sample surface on the XRD pattern. As shown in R1, the BP-Al shows a slight shift to a lower two-theta degree compared to BP, indicating the expanded interlayer distance, which might be related to the intercalated Al-related species between the interlayer of the BP nanosheet.

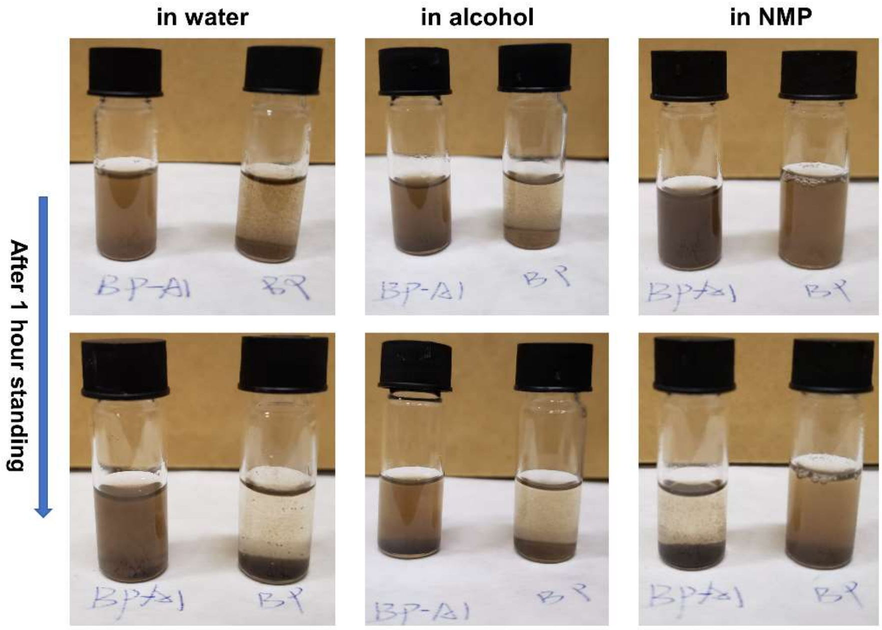

Figure S2. The dispersion stability of AlO_x-modified BP nanosheets (BP-Al) and pure BP nanosheets (BP) are compared in water, alcohol and NMP, respectively.

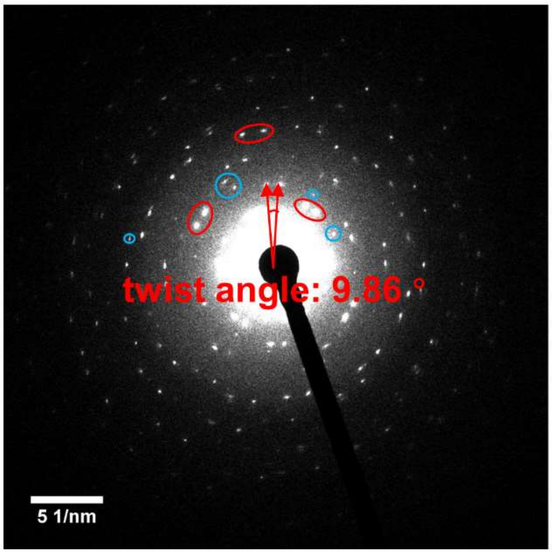

Figure S3. SAED images of the mixture of twisted and non-twisted diffraction spots.(red: twisted; blue: non-twisted).

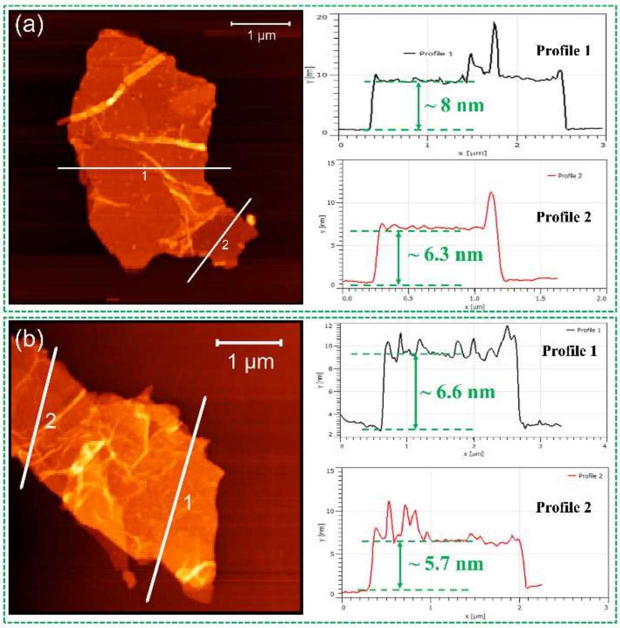

Figure S4 The corresponding thickness profiles of (a) BP-Al and (b) BP for time-resolved AFM analysis.

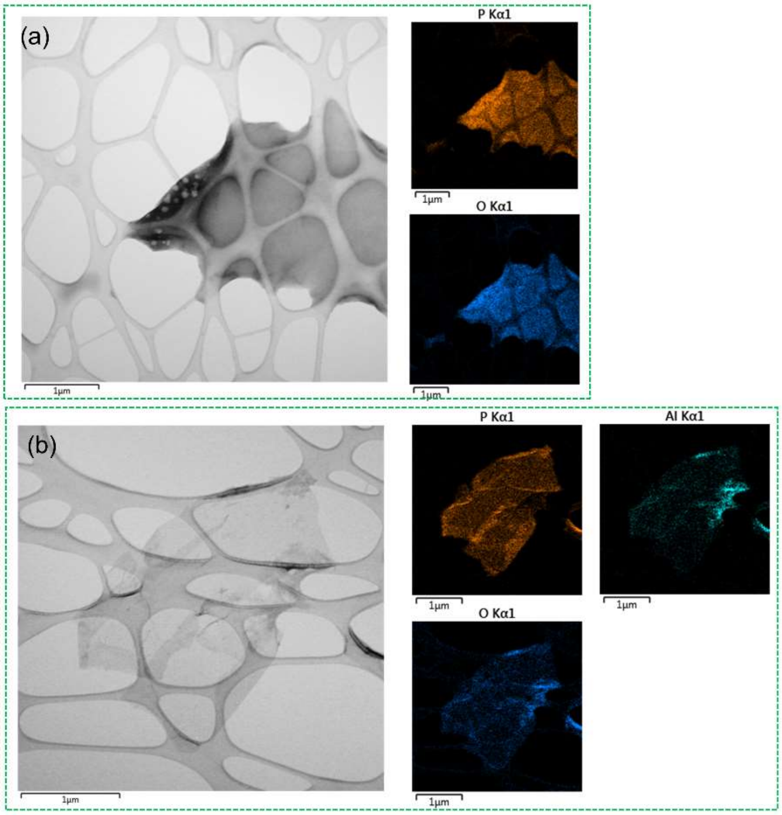

Figure S5 STEM-EDS results of (a) BP and (b) BP-Al after two-week exposure to the ambient condition.

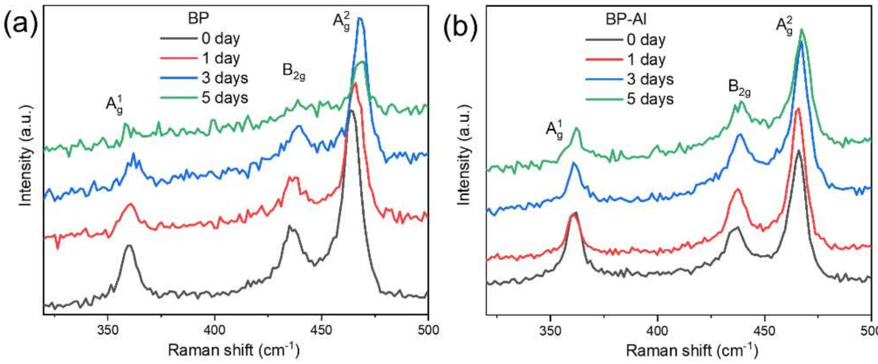

Figure S6 Time-dependent Raman spectrums of (a) BP and (b) BP-Al in 0, 1, 3, and 5 days under the ambient condition.
